# Supplementary material for: A paracrine activin A–mDia2 axis promotes squamous carcinogenesis via fibroblast reprogramming
Source: EMBO Mol Med. 2020 Mar 9;12(4):e11466. doi: 10.15252/emmm.201911466 (PMC7136968; doi:10.15252/emmm.201911466)
Supplement: Supplementary file 1 — Appendix [file EMMM-12-e11466-s001.pdf]

A paracrine activin A–mDia2 axis promotes squamous carcinogenesis via fibroblast reprogramming

Michael Cangkrama[1]*, Mateusz Wietecha[1], Nicolas Mathis[1], Rin Okumura[1], Luca Ferrarese[1], Dunja Al-Nuaimi[1], Maria Antsiferova[1,2], Reinhard Dummer[3], Metello Innocenti[4], and Sabine Werner[1]*

Appendix: Supplementary Information

Appendix Figure S1–S3

Appendix Table S1–S5

Appendix Fig S1.

A

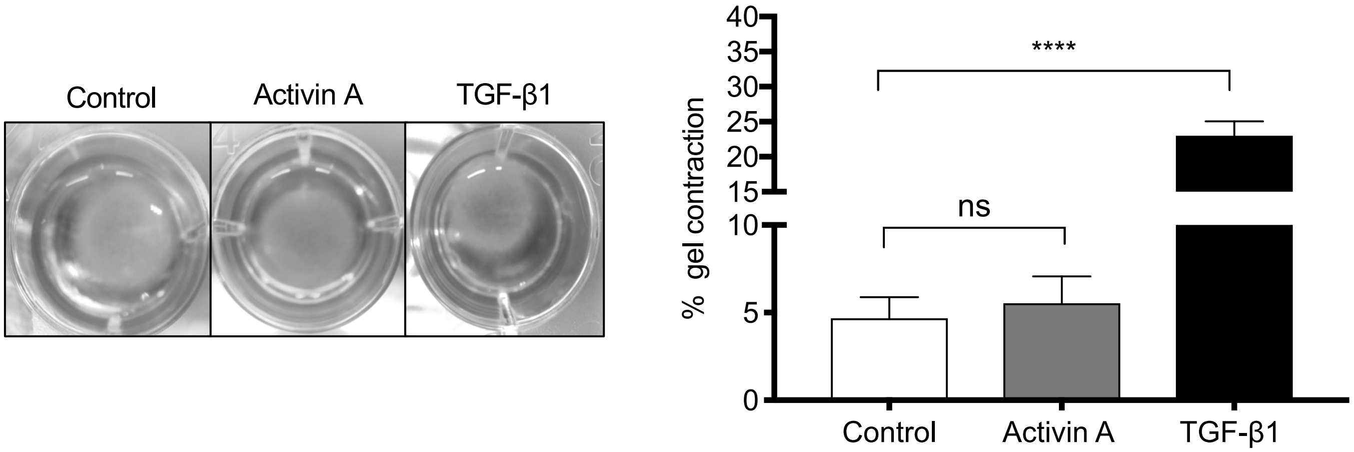

B

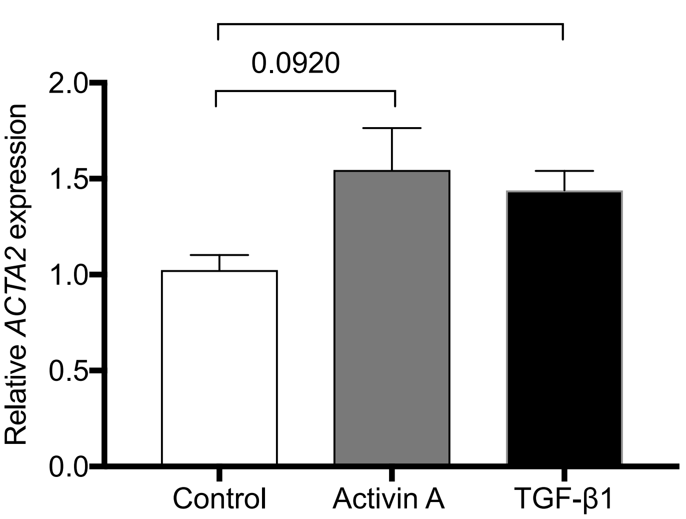

Appendix Fig S1 Activin A does not promote myofibroblast differentiation

A Representative images of collagen gels with embedded primary human dermal fibroblasts and quantification of the extent of gel contraction upon treatment with activin A (20 ng/ml) or TGF-β1 (1 ng/ml). N=3.

B qRT-PCR analysis for *ACTA2* relative to *RPL27* using RNA from fibroblasts treated with activin A or TGF-β1 for 6 h. N=3.

Bar graphs show mean +/- SEM. ns $p>0.05$, ****$p<0.0001$, one-way ANOVA with Bonferroni post-hoc test.

Appendix Fig S2.

A

IPA Upstream Regulator Analysis: **TP53**

Comparison	Activation Z-score	–Log10 (p-value)
Act/WT vs WT/WT	-2.98	11.08
Act/HPV8 vs WT/HPV8	-2.52	10.63
Act/HPV8 vs WT/WT	-3.78	10.72

Appendix Fig S2 Negative correlation between *INHBA* and p53 target gene expression in FACS-isolated fibroblasts from Act *vs*. wt mice

A Ingenuity pathway analysis of differentially expressed genes in FACS-isolated CD45^{-} CD140a^{+} fibroblasts from activin A-overexpressing compared to control mice, showing enrichment score of p53 target genes. Negative Z-scores predict that activin A reduces the expression of p53-target genes.

Appendix Fig. S3.

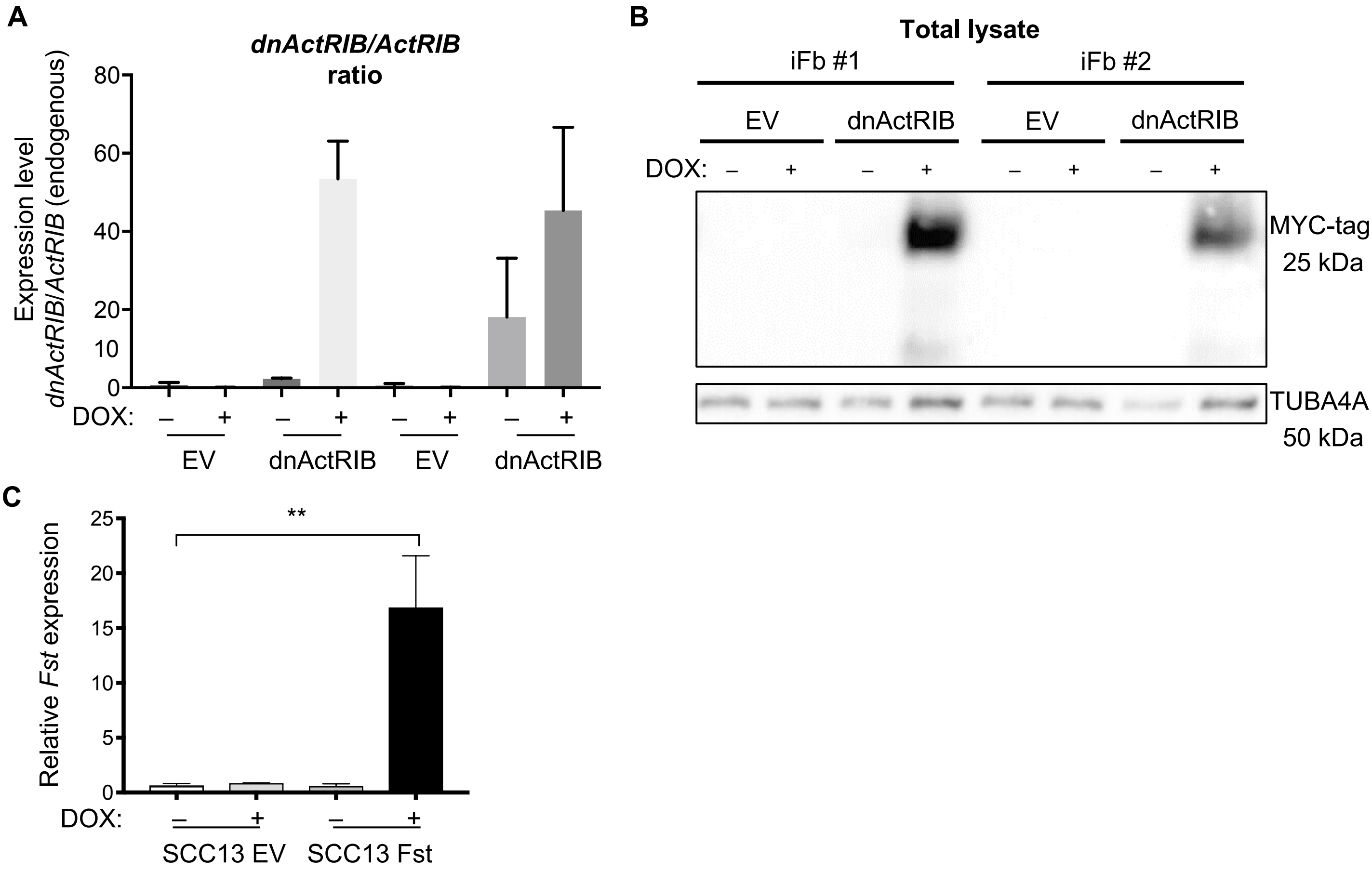

Appendix Figure S3 Expression of a dominant-negative type I activin receptor (dnActRIB) mutant or of follistatin in immortalized fibroblasts from PDGFRα-eGFP mice or in SCC13 cells, respectively.

A Ratio of dnActRIB *vs.* endogenous ActRIB mRNA in transduced fibroblasts from PDGFRα-eGFP mice (each normalized to *Rps29*) with and without DOX treatment (N=3).

B Western blot analysis of total lysate from fibroblasts transduced with EV or dnActRIB lentiviruses with an antibody detecting the Myc epitope in the dnActRIB-Myc fusion protein.

C qRT-PCR for *Fst* relative to *RPL27* using RNA from SCC13 cells transduced with a lentiviral vector expressing murine Fst in a DOX-inducible manner or EV. N=3. Bar graphs show mean and SEM. ** $p<0.01$. one-way ANOVA with Bonferroni post-hoc test.

Appendix Table S1: Primer sequences for amplification of fragments required for cloning

**Gene Name**	**Forward primer (5'-3')**	**Reverse primer (5'-3')**
*INHBA**	CAC CAT GCC CTT GCT TTG	CTA TGA GCA CCC ACA CTC CTC C
FST	CAC CAT GTA CCC ATA CGA TGT TCC AGA TTA CGC TGT CTG CGC CAG GCA CC	TTA CCA CTC TAG AAT GGA AGA GAT AGG AAA GCT GTA GT
dnActRI†	CAC CAT GGC GGA GTC GGC CGG AGC CTC	TTA CAG GTC TTC TTC AGA GAT CAG TTT CTG TTC

(an in-frame *HA-tag or †MYC-tag sequence was fused in-frame at the 3' end of the coding sequence)

Appendix Table S2: shRNA sequences

**shRNA mDia2**	**Sequence (5'-3')**
sh-mDia2 #1 (TRCN0000108778)	CCGGGCAGAGAAAGAGCGACTTGAACTCGAGTTCAAGTCG CTCTTTCTCTGCTTTTTG
sh-mDia2 #2 (TRCN0000108779)	CCGGCGACCCAAGTTGCATTTAAATCTCGAGATTTAAATGC AACTTGGGTCGTTTTTG
sh-mDia2 #3 (TRCN0000108777)	CCGGCCCTGACTTCACATACAGAAACTCGAGTTTCTGTATG TGAAGTCAGGGTTTTTG
sh-mDia2-human (TRCN0000150903)	GCTCAGTGCTATTCTCTTTAA

Appendix Table S3: Primer sequences for qRT-PCR

	**Forward primer (5'-3')**	**Reverse primer (5'-3')**
Acta2	TCC CTG GAG AAG AGC TAC GA	CTT CTG CAT CCT GTC AGC AA
ACTA2	GTG ACG AAG CAC AGA GCA AA	GAG TCA TTT TCT CCC GGT TG
Inhba	GGA GAA CGG GTA TGT GGA GA	ACA GGT CAC TGC CTT CCT TG
INHBA	CCT CGG AGA TCA TCA CGT TT	CCC TTT AAG CCC ACT TCC TC
INHBB	AGC TTC GCC GAG ACA GAT G	CGT AGG GCA GGA GTT TCA GG

INHA	CCA GCT GTG AGG ACA AGT CA	CTA GCA GGG GCT CAG AGC TA
mDia1	GGA TCC TGC TGT TCC CAA TA	CCC ACT TTT TAA TCC GTC CA
mDia2	TTT GCA TAG ATC AGGCCA AAC	GCT TGT AAC TCT GCT TGA AGC TC
DIAPH3	GCG GGA AAA GGA CTT CAG TA	TTG CTG GTC AAA GAC ACT CG
mDia3	AGC AAT TGA CCC GAA ACA AC	TCC ACA ATT GGT GAA AAT CG
Fst	AGG GAA AGT GTA TCA CAA AGT	GAG TTG CAA GAT CCA GAA TG
FST	CGT GAA TGA CAA CAC ACT CTT C	TTT TTC CCA GGT CCA CAG TC
TGFB1	QT00000728 (Forward + Reverse) (QuantiTect, Qiagen)	
Postn	AAG ACT GCT TCA GGG AGA CAC A	TCA GTG TGG TGG CTC TTA CA
POSTN	CAG CCT TTC ATT CCT TCC AT	CAA ATG TCT GTG CCC TTC AA
Spp1	CCA GCA GCT CAC ACT GAA GA	CCA AAC AGG CAA AAG CAA AT
Mmp13	ATC CTG GCC ACC TTC TTC TT	TTT CTC GGA GCC TGT CAA CT
Fn1	TGT GAC AAC TGC CGT AGA CC	GAC CAA CTG TCA CCA TTG AGG
FN1	GCA GGT ACA GTC CCA GAT CA	AGC GGA CCT ACC TAG GCA AT

Trp53	TTT TGA AGG CCC AAG TGA AG	TGA GGG GAG GAG AGT ACG TG
Il6	CCG GAG AGG AGA CTT CAC AG	TTC TGC AAG TGC ATC ATC GT
Serpine1	CGC CTC CTC ATC CTG CCT AAG	CTG TGC CGC TCT CGT TTA CC
SERPINE1	CAT CAT GGG CAC AGA GAC AG	GGT GGA GAG AGC CAG ATT CA
Fmn1	ATA AGG ATG GAG GGG TTT GG	CTT GGA GTC TGC CTG GAG TC
Fmn2	AAC CCA CAC ATT CGA TGG AT	CTG GAC ACC ATT TGA GCA TTT
Alk4	GGG TGG GGA CCA AAC GAT AC	GAG GGC ATA GAT GTC GGC AC
dnAlk4-myc	TCA TCG TCT TCC TGG TCA TCA A	ATC AGT TTC TGT TCC CCA CCA C
Rbp-Jκ (Csl)	ACG CCA GTT CAC AAC AGT G	GTC TGC CCG TAA TGG ATG TA
RPL27	TCA CCT AAT GCC CAC AAG GTA	CCA CTT GTT CTT GCC TGT CTT
Rps29	GGT CAC CAG CAG CTC TAC TG	GTC CAA CTT AAT GAA GCC TAT GTC C

Appendix Table S4: List of antibodies and dyes used for fluorescence/ immunofluorescence staining

Name (Prod. No.)	Company	Dilution
Alexa Fluor® 488 AffiniPure donkey anti-mouse IgG (H+L) (#715-545-150)	Jackson ImmunoResearch	1:200
Alexa Fluor® 594 AffiniPure donkey anti-rabbit IgG (H+L) (#711-587-003)	Jackson ImmunoResearch	1:200
Alexa Fluor™ 555 phalloidin (#A34055)	Thermo Fisher Scientific	1:200
Chicken anti-β-catenin (C7207)	Sigma-Aldrich	1:1000
Cy™2 AffiniPure donkey anti-goat IgG (H+L) (#705-225-147)	Jackson ImmunoResearch	1:200
Cy™3 AffiniPure donkey anti-rabbit IgG (H+L) (#711-165-152)	Jackson ImmunoResearch	1:200
Cy™3 AffiniPure donkey anti-chicken IgG (H+L) (#703-165-155)	Jackson ImmunoResearch	1:200
Hoechst 33342 (B2261)	Sigma-Aldrich	1:1000
Mouse anti-E-cadherin (#610181)	BD Pharmingen	1:100
Mouse anti-SMAD2/3 (C-8) (sc-133098)	Santa Cruz	1:500

Mouse anti-α-SMA (A2547)	Sigma-Aldrich	1:800
Mouse anti-α-SMA FITC (F3777)	Sigma-Aldrich	1:1000
Mouse anti-p53 (sc-126)	Santa Cruz	1:500
Rabbit anti-DIAPH3 (H-42) (sc-135261)	Santa Cruz	1:1000
Rabbit anti-keratin 14 (#PRB-155P)	Biolegend	1:5000
Rabbit anti-LYVE1 (#11034)	Angiobio	1:500
Rat anti-MECA32 (#553849)	BD Pharmingen	1:500
Goat anti-PDGFR-α (#AF1062)	R&D Systems	1:500
Mouse anti-collagen IV (#10710)	Progen	1:500

Appendix Table S5: Primer sequences for ChIP

**Gene Name**	**Forward primer (5'-3')**	**Reverse primer (5'-3')**
mDia2 (Intron 1)	CTGGAGGAGGATGTGTCCAT	AGCCAAGGCACAAAGGACTA
