# Supplementary material for: A paracrine activin A–mDia2 axis promotes squamous carcinogenesis via fibroblast reprogramming
Source: EMBO Mol Med. 2020 Mar 9;12(4):e11466. doi: 10.15252/emmm.201911466 (PMC7136968; doi:10.15252/emmm.201911466)
Supplement: Supplementary file 2 — Expanded View Figures PDF [file EMMM-12-e11466-s002.pdf]

Expanded View Figures

Figure EV1. Characterization of SCC cells overexpressing activin A in monoculture and *in vivo*.

A qRT–PCR for *INHBB*, *INHA*, *TGFB1*, and *FST* relative to *RPL27* using RNA from SCC13 cells transduced with a lentiviral vector expressing *INHBA* in a DOX-inducible manner (N = 3).

B Left panel: Total cell number of exponentially growing SCC13 EV and SCC13 Act cells at different time points after seeding. N = 3. Right panel: Migration in scratch assays of SCC13 EV and SCC13 Act cells (clones 1 and 2) (N = 3–4). Migration of one control culture was set to 1.

C Representative images of PFA-fixed SCC13 cells stained for E-cadherin and β-catenin, K14, and total SMAD2/3 as indicated and counterstaining of nuclei with Hoechst.

D qRT–PCR for *INHBA* relative to *RPL27* using RNA from A431 cells transduced with a lentiviral vector expressing *INHBA* in a DOX-inducible manner or EV (N = 3).

E Western blot of total lysate of transduced A431 cells using antibodies against the activin βA subunit and GAPDH.

F Representative pictures of 5-week-old tumors formed upon intradermal injection of A431 Act or EV cells into the ear skin of NOD/SCID mice.

G, H Tumor volume at different time points of tumor development (G) and tumor weight 5 weeks after injection (H) (N = 5 tumors).

I Immunofluorescence staining of serial sections from ear skin tumors formed by SCC13 EV or SCC13 Act cells 5 weeks after injection for K14, E-cadherin, and β-catenin (green) combined with Hoechst staining of nuclei (blue). The square in the upper panel indicates the area that is shown at higher magnification in the lower panel.

J, K Representative images of tumor sections stained for MECA32 (red) and K14 (green) (J) or LYVE1 (red) (K) combined with Hoechst staining (blue) and quantification of the positive area using ImageJ. N = 3 tumors, n = 3–4 histological sections.

Data information: Bar graphs show mean ± SEM. ns = non-significant; *$P > 0.05$ (unpaired Student's t-test (H, J, K), one-way ANOVA with Bonferroni post hoc test (A, B (right panel), D), and two-way ANOVA with Bonferroni post hoc test (B (left panel), G). Scale bars: 100 μm.

Source data are available online for this figure.

 © 2020 The Authors

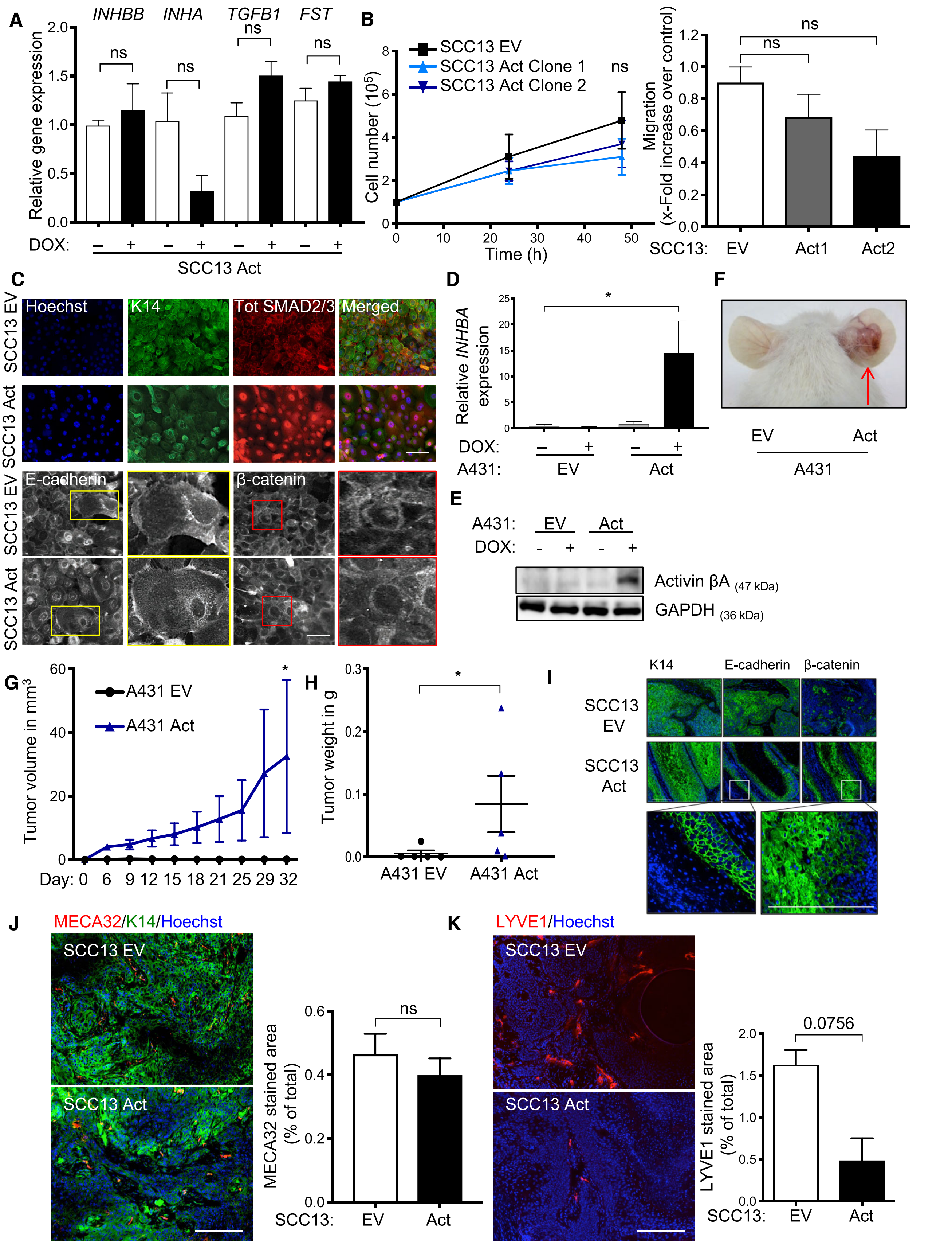
A
INHBB INHA TGFB1 FST
ns ns ns ns
Relative gene expression
DOX: – + – + – + – +
SCC13 Act
B
SCC13 EV
SCC13 Act Clone 1
SCC13 Act Clone 2
ns
Cell number (10^5)
Time (h)
Migration (x-Fold increase over control)
SCC13: EV Act1 Act2
C
Hoechst K14 Tot SMAD2/3 Merged
SCC13 EV
SCC13 Act
E-cadherin
β-catenin
D
Relative INHBA expression
DOX: – + – +
A431: EV Act
F
EV Act
A431
E
A431: EV Act
DOX: - + - +
Activin βA (47 kDa)
GAPDH (36 kDa)
G
A431 EV
A431 Act
Tumor volume in mm3
Day: 0 6 9 12 15 18 21 25 29 32
H
Tumor weight in g
A431 EV A431 Act
I
K14 E-cadherin β-catenin
SCC13 EV
SCC13 Act
J
MECA32/K14/Hoechst
SCC13 EV
SCC13 Act
MECA32 stained area (% of total)
SCC13: EV Act
K
LYVE1/Hoechst
SCC13 EV
SCC13 Act
0.0756
LYVE1 stained area (% of total)
SCC13: EV Act

Figure EV1.

Figure EV2. Activin A promotes invasive growth of cancer cells in organotypic cultures.

A Representative images of H&E and Herovici stainings of A431 EV and Act cells cultured in 3D with primary human fibroblasts.

B Herovici staining of SCC13 EV and SCC13 Act cells (clone 2) cultured in 3D with primary fibroblasts from wild-type mice or immortalized fibroblasts from PDGFRα-eGFP transgenic mice. Lowest panel shows eGFP-positive fibroblasts (green), counterstained with Hoechst (blue).

C, D Representative images of sections from SCC13 EV and SCC13 Act (clone 2) organotypic cultures stained for E-cadherin (C) and β-catenin (D). Red asterisks indicate cells with cyto-nuclear β-catenin. The rectangles in the upper panel indicate the area shown at higher magnification in the lower panel.

E Gelatin zymography analysis of conditioned media from A431 Act and A431 EV (upper panel) or SCC13 EV and SCC13 Act (Clone 2) (lower panel) organotypic cultures. $N = 2–3$.

Data information: Bar graphs show mean ± SEM. ns $P > 0.05$, *$P < 0.05$, **$P < 0.01$, ***$P < 0.001$, ****$P < 0.0001$ (unpaired Student's t-test (E)). Scale bars (A-D): 100 μm.

© 2020 The Authors

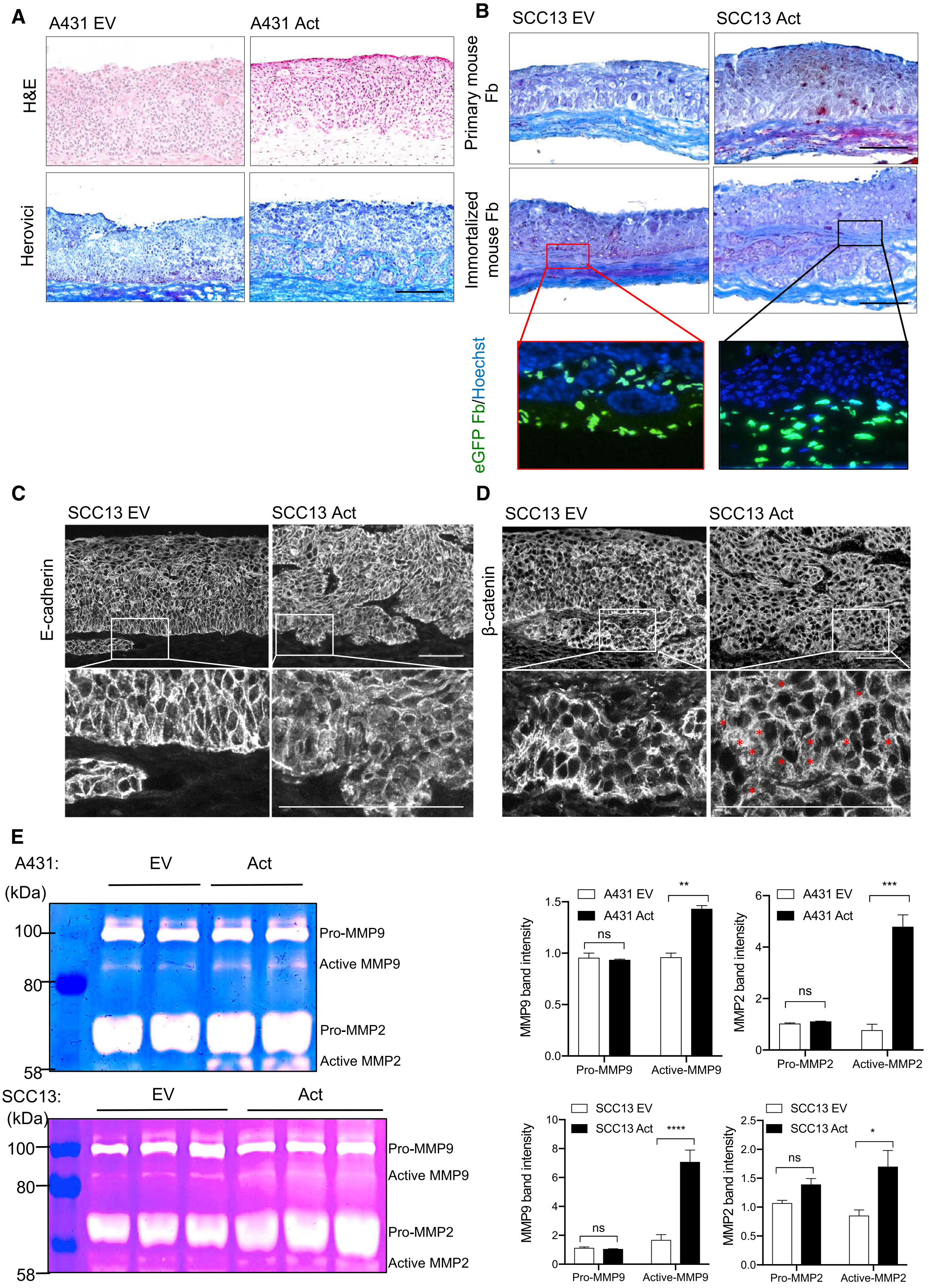

A
A431 EV
A431 Act
H&E
Herovici
B
SCC13 EV
SCC13 Act
Primary mouse Fb
Immortalized mouse Fb
eGFP Fb/Hoechst
C
SCC13 EV
SCC13 Act
E-cadherin
D
SCC13 EV
SCC13 Act
β-catenin
E
A431:
EV
Act
(kDa)
100
80
58
Pro-MMP9
Active MMP9
Pro-MMP2
Active MMP2
SCC13:
EV
Act
(kDa)
100
80
58
Pro-MMP9
Active MMP9
Pro-MMP2
Active MMP2
A431 EV
A431 Act
MMP9 band intensity
Pro-MMP9
Active-MMP9
ns
**
MMP2 band intensity
Pro-MMP2
Active-MMP2
ns
***
SCC13 EV
SCC13 Act
MMP9 band intensity
Pro-MMP9
Active-MMP9
ns
****
MMP2 band intensity
Pro-MMP2
Active-MMP2
ns
*

Figure EV2.

Figure EV3. RNA sequencing of FACS-isolated fibroblasts from ear skin of control and activin A-overexpressing mice.

A Scheme of the experimental setup for RNA sequencing of mouse ear skin fibroblasts. Ear skin from 3 to 6 mice per genotype was pooled, and $CD45^{+}CD140a^{-}$ fibroblasts were isolated by FACS and subjected to RNA sequencing. *N* = 3.
B Re-analysis of the sorted cells by flow cytometry showing a purity above 90%.
C Analysis of FACS data of sorted fibroblasts showing the relative percentage of $CD45^{-}CD140a^{+}$ cells in mice of each genotype and the absolute number of sorted cells used for RNA sequencing. *N* = 5–9.
D Expression of fibroblast and non-fibroblast markers based on RNA sequencing data.
E Number of differentially expressed genes (FDR < 0.05, $|\log_2 FC| > 1$) identified by RNA sequencing. Venn diagram shows *mDia2* as one of the top regulated genes.
F qRT–PCR analysis for *mDia2/DIAPH3* relative to *RPL27* using RNA from human fibroblasts treated with activin A or TGF-β1 for 6. *N* = 3.
G *mDia1* and *mDia3* expression quantified by sequencing of RNA from fibroblasts isolated from ear skin. *N* = 3 pools of 3–6 mice.
H qRT–PCR for *mDia1* and *mDia3* relative to *Rps29* using RNA from fibroblasts treated with activin A for 6 h. *N* = 3.
I GTRD database analysis predicting SMAD2/3 binding sites within 100 kb upstream of the transcription start site (TSS) or within the genomic region of the human *mDia1-3*/*DIAPH1-3* gene.

Data information: Graphs show mean ± SEM. ns = non-significant; *$P < 0.05$, **$P < 0.01$. Unpaired Student's *t*-test (C, H) and one-way ANOVA with Bonferroni post hoc test (F, G).

 © 2020 The Authors

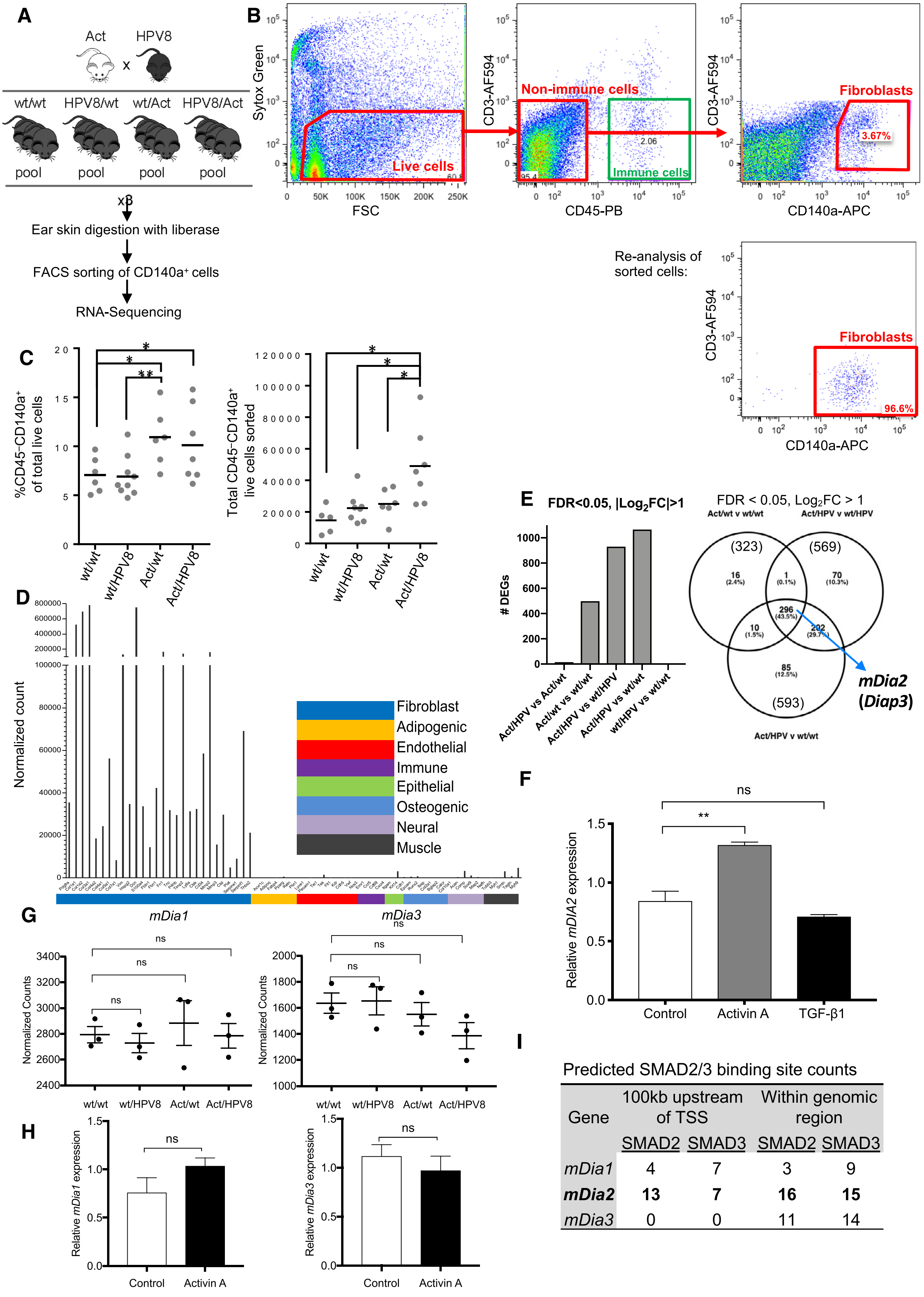

Predicted SMAD2/3 binding site counts

Gene	100kb upstream of TSS		Within genomic region	
	SMAD2	SMAD3	SMAD2	SMAD3
mDia1	4	7	3	9
mDia2	**13**	**7**	**16**	**15**
mDia3	0	0	11	14

Figure EV3.

Figure EV4. Characterization of mDia2 knock-down in mouse (sh-mDia2 #2) and human fibroblasts.

A, B Percentage of Ki67-positive cells (A) and migration in scratch assays (B) of mDia2 knock-down and control immortalized mouse fibroblasts. $N = 3$.

C qRT–PCR analysis for *mDia2/DIAPH3* relative to *RPL27* using RNA from mDia2 knock-down and control primary human skin fibroblasts. $N = 3$. Western blot of total lysate of mDia2 knock-down and control human fibroblasts for mDia2 (DIAPH3) and α-tubulin.

D, E BrdU incorporation (D) and migration (E) in scratch wounds of mDia2 knock-down and control primary human skin fibroblasts. $N = 3$.

F Primary human skin fibroblasts were transfected with an expression vector encoding murine mDia2 (pMX-mDia2) or empty vector (pMX-control), and expression of CAF markers, including *INHBA* and *DIAPH3*, relative to *RPL27* was analyzed by qRT–PCR at 48 h post-transfection. $N = 3$.

G Relative colony area of SCC13 cells after plating on de-cellularized matrix deposited by primary human fibroblasts, which had been transfected with a pMX-mDia2 expression vector or empty vector. $N = 6$.

H Relative colony area of SCC13 cells after incubation in conditioned medium (CM) from pMX-mDia2 or pMX-transfected fibroblasts. $N = 6$.

I Quantification of nuclear p53 in mDia2 knock-down and control primary human skin fibroblasts. $N = 3$.

J, K Western blot analysis of p53, the cytoplasmic marker vinculin, and the nuclear marker histone H1 using nuclear and cytoplasmic fractions and total lysate of primary human skin fibroblasts and quantification of the p53/histone H1 ratio in the nucleus. $N = 3$.

L Immunoprecipitation using anti-DIAPH3 antibodies or control IgG and lysates of primary human fibroblasts treated with activin A (20 ng/ml) or vehicle, followed by Western blot analysis for mDia2, p53, and β-actin (loading control). Note the presence of p53 in the mDia2 immunoprecipitate, in particular in activin A-treated cells.

M Colony size of SCC13 Act cells after co-culture with sh-mDia2 knock-down human fibroblasts or control fibroblasts transduced with EV. $N = 3$.

N Western blot analysis of total lysate from INHBA-overexpressing human fibroblasts with knock-down of mDia2.

O Relative colony area of SCC13 cells after plating on de-cellularized matrix deposited by Fb Act or Fb EV. $N = 3$.

P Relative colony area of SCC13 cells after incubation in CM from Fb Act or Fb EV. $N = 3$.

Data information: Bar graphs show mean and SEM. ns $P > 0.05$, *$P < 0.05$, **$P < 0.01$, ****$P < 0.0001$. Unpaired Student's *t*-test (A–K, M) and one-way ANOVA with Bonferroni post hoc test (O–P).

Source data are available online for this figure.

© 2020 The Authors

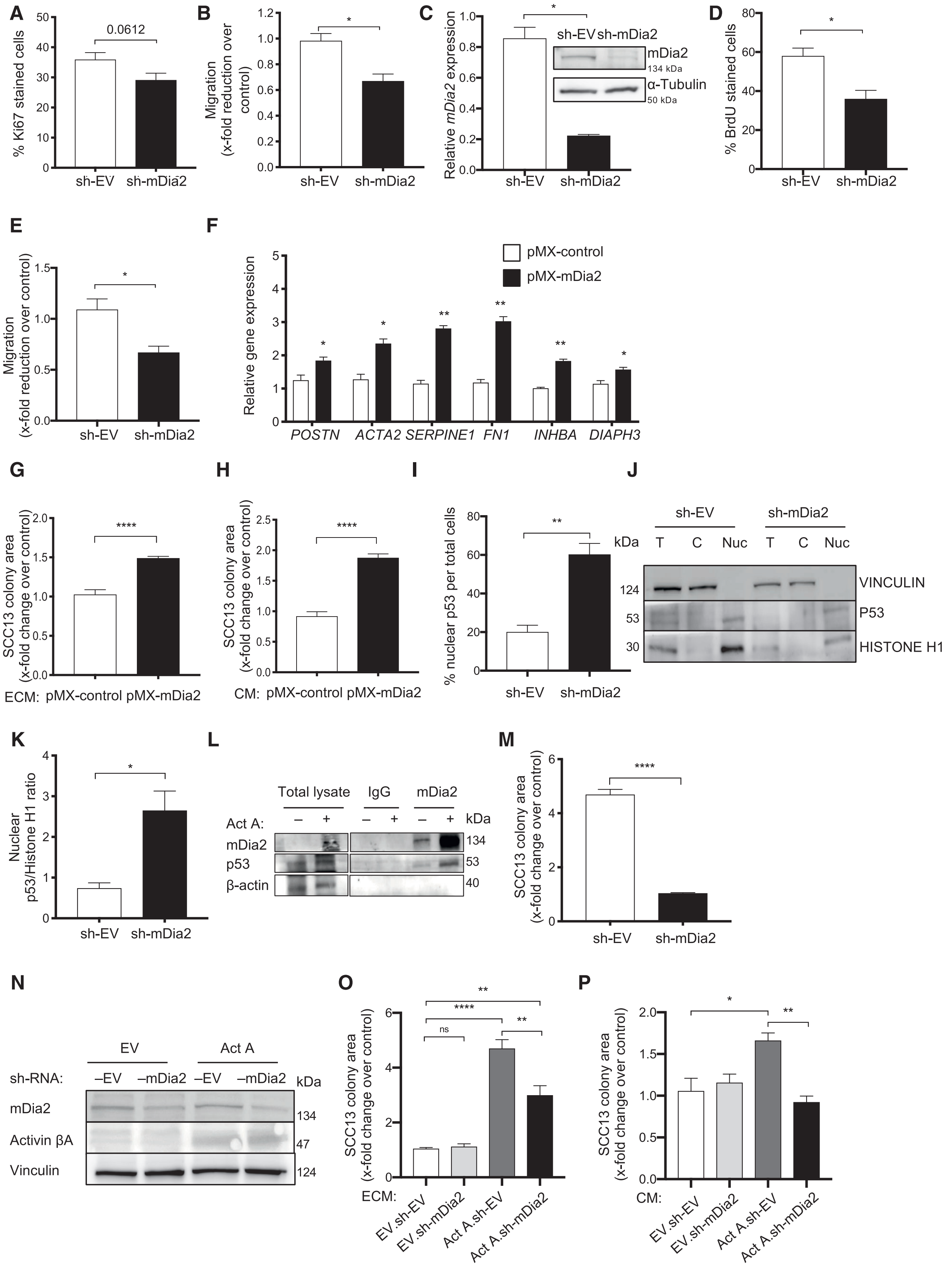
A
% Ki67 stained cells
0.0612
sh-EV sh-mDia2
B
Migration (x-fold reduction over control)
sh-EV sh-mDia2
C
Relative mDia2 expression
sh-EV sh-mDia2
mDia2
134 kDa
α-Tubulin
50 kDa
D
% BrdU stained cells
E
Migration (x-fold reduction over control)
F
Relative gene expression
pMX-control
pMX-mDia2
POSTN ACTA2 SERPINE1 FN1 INHBA DIAPH3
G
SCC13 colony area (x-fold change over control)
ECM: pMX-control pMX-mDia2
H
CM: pMX-control pMX-mDia2
I
% nuclear p53 per total cells
J
sh-EV sh-mDia2
kDa T C Nuc T C Nuc
VINCULIN
P53
HISTONE H1
K
Nuclear p53/Histone H1 ratio
L
Total lysate IgG mDia2
Act A:
kDa
mDia2
p53
β-actin
M
N
EV Act A
sh-RNA: –EV –mDia2 –EV –mDia2 kDa
mDia2
Activin βA
Vinculin
O
ECM: EV.sh-EV EV.sh-mDia2 Act A.sh-EV Act A.sh-mDia2
P
CM: EV.sh-EV EV.sh-mDia2 Act A.sh-EV Act A.sh-mDia2

Figure EV4.
