# Supplementary figures and images for: A paracrine activin A–mDia2 axis promotes squamous carcinogenesis via fibroblast reprogramming
Source: EMBO Mol Med. 2020 Mar 9;12(4):e11466. doi: 10.15252/emmm.201911466 (PMC7136968; doi:10.15252/emmm.201911466)

Fig Appendix S3:

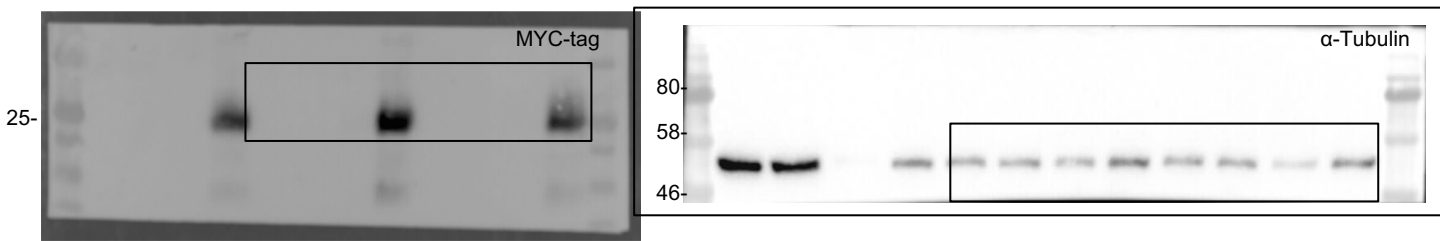

Supplement: Supplementary file 6 — Source Data for Expanded View and Appendix [file EMMM-12-e11466-s011.zip › EMM_11466_Source_Data_App_Fig_S3.pdf]

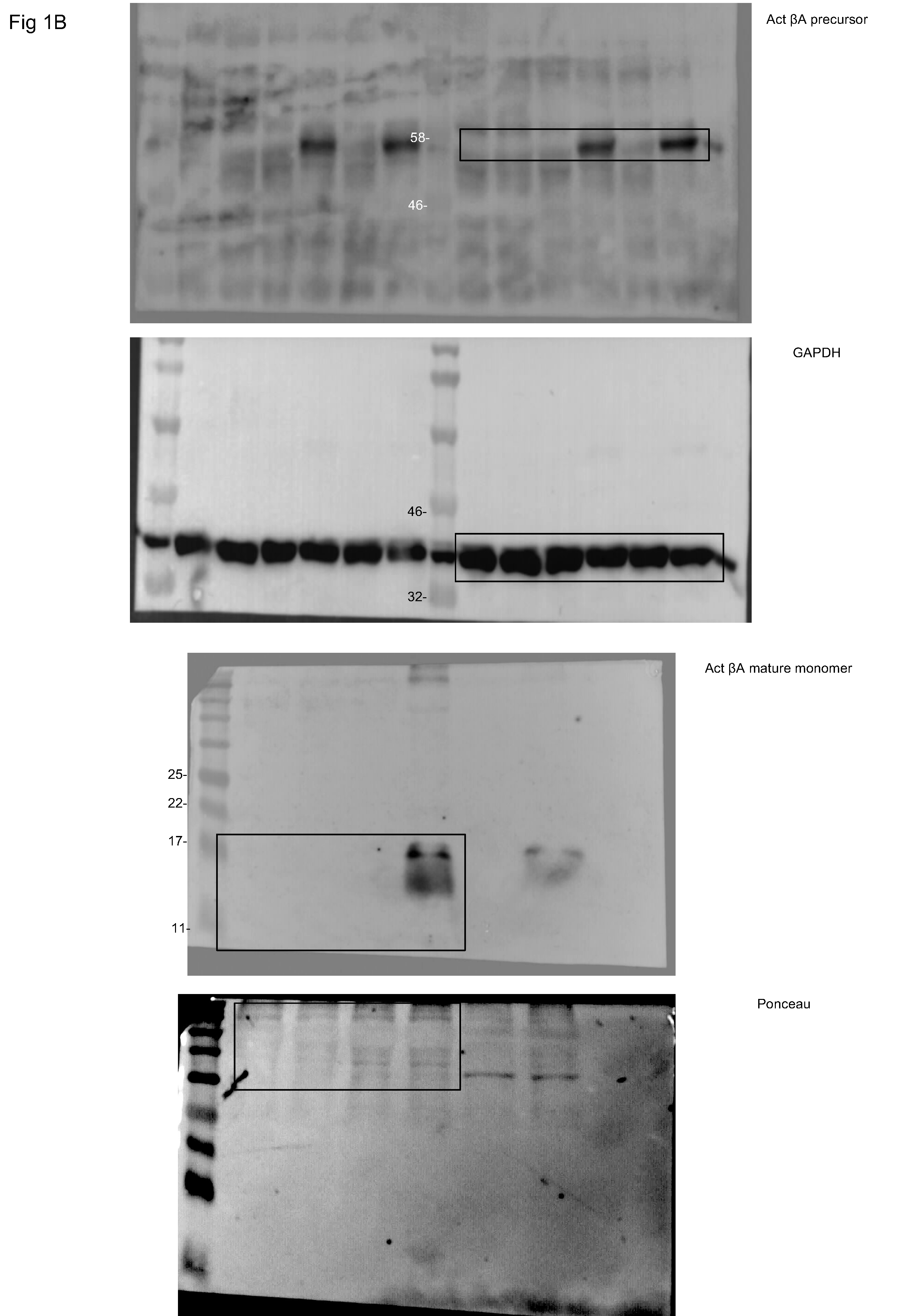

Supplement: Supplementary file 8 — Source Data for Figure 1 [file EMMM-12-e11466-s006.tiff]

Fig 2C

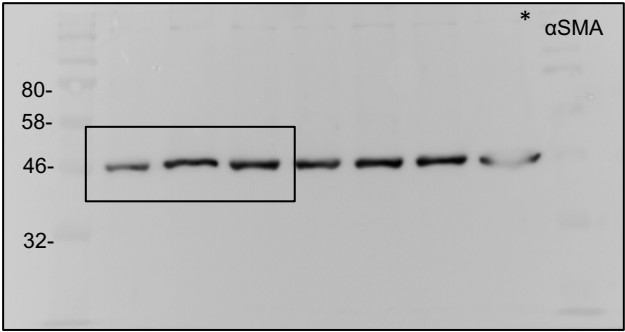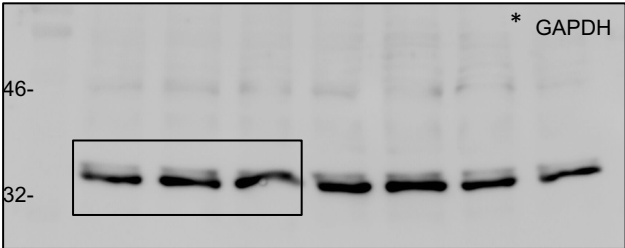

Supplement: Supplementary file 9 — Source Data for Figure 2 [file EMMM-12-e11466-s007.pdf]

Fig 3B

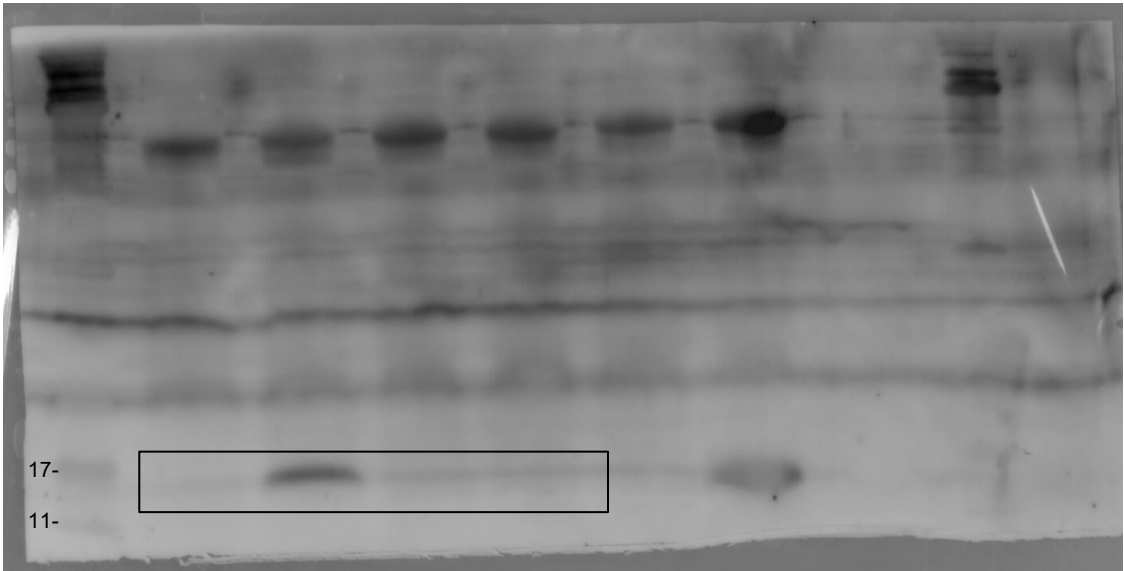

Ponceau S

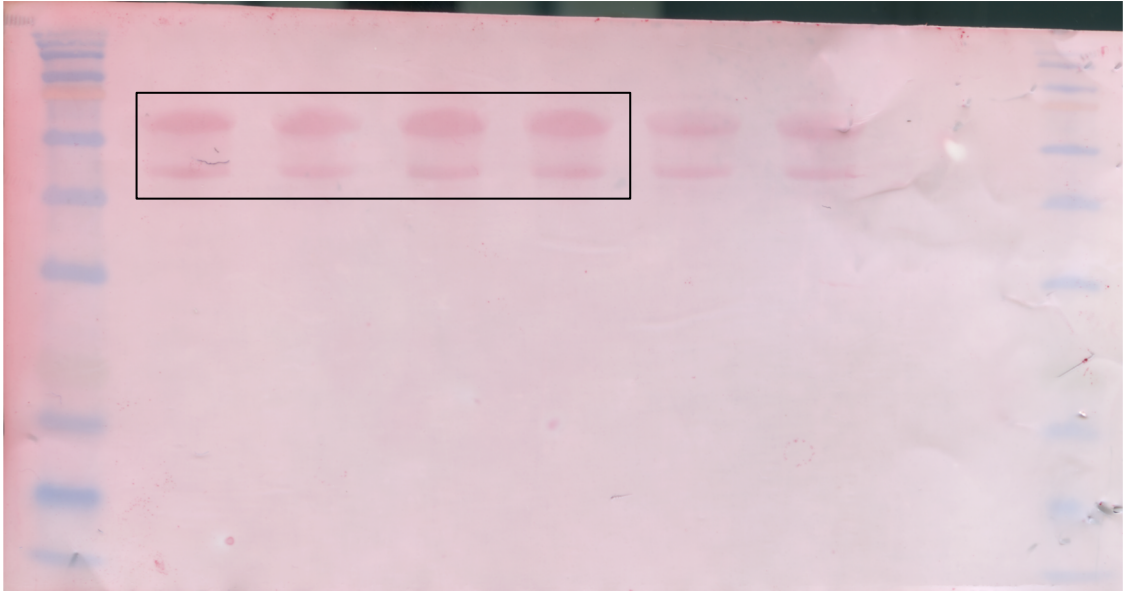

Supplement: Supplementary file 10 — Source Data for Figure 3 [file EMMM-12-e11466-s008.pdf]

Fig 4E

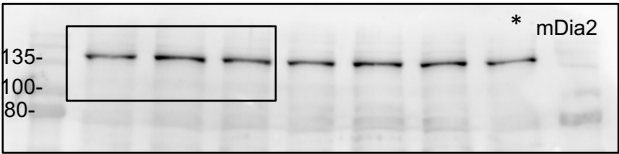

\* Same blot, re-probed with  $\alpha$ SMA, mDia2 and GAPDH

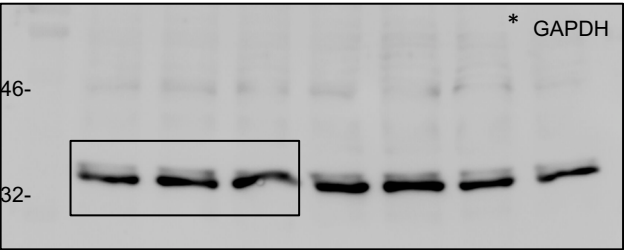

Fig 4F

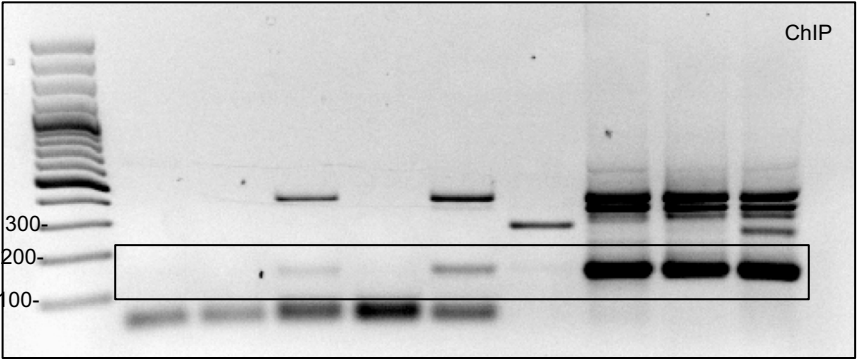

Expected PCR product: 190 bp

Fig 4G

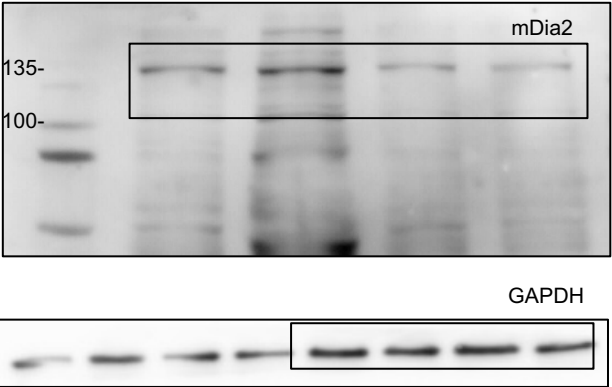

Supplement: Supplementary file 11 — Source Data for Figure 4 [file EMMM-12-e11466-s009.pdf]

Fig 6A

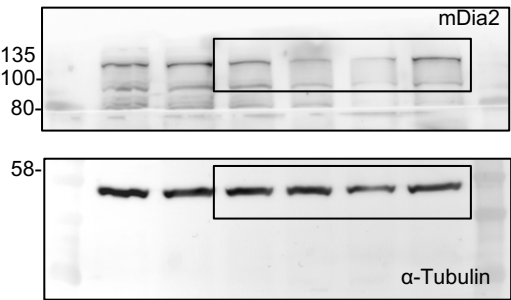

Fig 6F

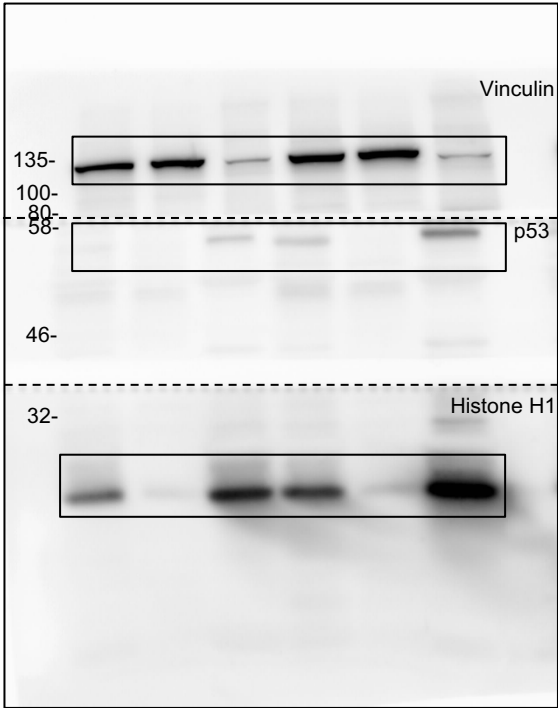

Fig 6G

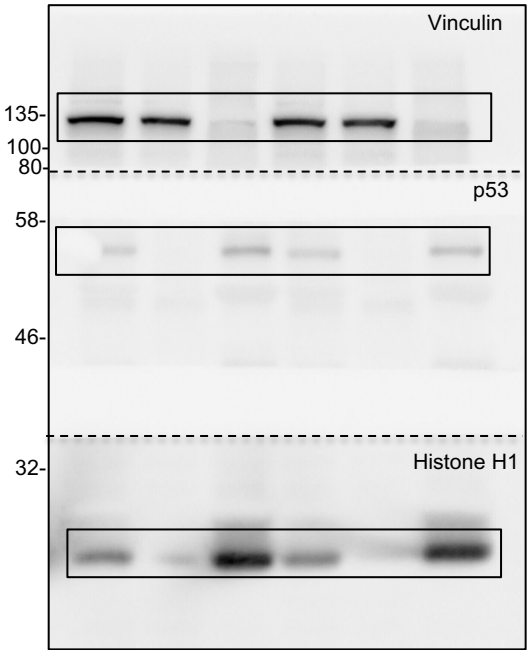

Fig 6H

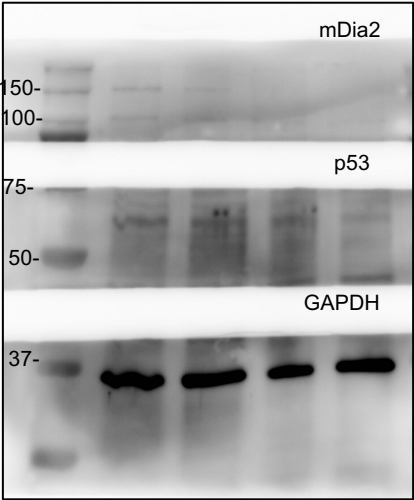

Supplement: Supplementary file 12 — Source Data for Figure 6 [file EMMM-12-e11466-s010.pdf]
