# Supplementary material for: A paracrine activin A–mDia2 axis promotes squamous carcinogenesis via fibroblast reprogramming
Source: EMBO Mol Med. 2020 Mar 9;12(4):e11466. doi: 10.15252/emmm.201911466 (PMC7136968; doi:10.15252/emmm.201911466)

Fig EV1E

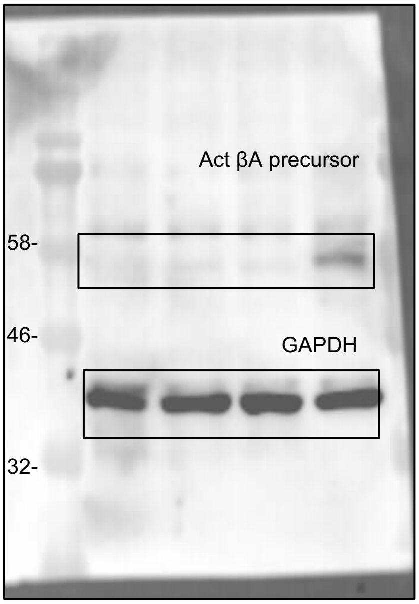

Supplement: Supplementary file 6 — Source Data for Expanded View and Appendix [file EMMM-12-e11466-s011.zip › Source_Data_EV_Fig.zip!/EMM_11466_Source_Data_Fig_EV1.pdf]
